# Rescue percutaneous transhepatic portal vein embolization after failed associated liver partition and portal vein ligation for staged hepatectomy in a patient with multiple liver metastases of rectal cancer: a case report

**DOI:** 10.1186/s40792-022-01491-w

**Published:** 2022-07-14

**Authors:** Hidenori Tomida, Tsuyhosi Notake, Akira Shimizu, Koji Kubota, Kentaro Umemura, Atsushi Kamachi, Takamune Goto, Shiori Yamazaki, Yuji Soejima

**Affiliations:** Division of Gastroenterological, Hepato-Biliary-Pancreatic, Transplantation and Pediatric Surgery, Department of Surgery, Shinshu University School of Medicine, 3-1-1 Asahi, Matsumoto, Nagano 390-8621 Japan

**Keywords:** Liver metastasis, ALPPS, PVE

## Abstract

**Background:**

Liver metastasis is the most common form of distant spread of colorectal cancer. Despite oncological and surgical advances, only about 25% of patients are eligible to undergo resection. As the liver has a limited resectable volume, tumor reduction and remnant liver hypertrophy are of critical importance in treating initially unresectable colorectal cancer liver metastasis. Associated liver partition and portal vein ligation for staged hepatectomy (ALPPS) allows rapid liver hypertrophy within a short period and has been reported to be useful in recent years.

**Case presentation:**

A 29-year-old woman complaining of bloody stool was referred to our hospital. She was diagnosed with rectal cancer (Rb) with simultaneous multiple liver and lung metastases. The patient was then initially commenced on chemotherapy and completed it with a satisfactory response. Right trisectionectomy was necessary to achieve hepatic clearance; however, the future liver remnant (FLR) volume was insufficient. Therefore, we decided to perform totally laparoscopic ALPPS to obtain enough FLR volume. However, the FLR increase was slow, and FLR did not attain the required volume for right trisectionectomy. Computed tomography showed that right portal venous blood flow was increased via developed collateral vessels around the portal vein. We attempted to induce further liver growth by blocking portal blood flow using additional percutaneous transhepatic portal vein embolization (PTPE), and a rapid increase in FLR was obtained. The patient underwent right trisectionectomy and partial resection of S2 with negative margins, and the patient was discharged without postoperative liver failure.

**Conclusions:**

Resumption of the portal venous blood flow through collateral vessels after ALPPS may have interfered with the planned residual liver hypertrophy. Performing PTPE in addition to ALPPS increased the FLR volume, and radical hepatectomy was completed safely. Remnant portal venous blood flow following ALPPS is an important issue to be considered in surgical planning, and early additional portal vein embolization could be effective.

## Background

Liver metastasis is the most common form of distant spread, affecting approximately 15–25% of patients with colorectal cancer (CRC) who have distant metastases at the time of primary diagnosis. Indications for curative treatment for colorectal cancer liver metastasis (CRCLM) have recently expanded.

Unfortunately, despite surgical and oncological advancements, approximately only 25% of patients are eligible for surgical resection, which is regarded as the only curative treatment for CRCLM. Numerous methods are still under development for inoperable cases. As the liver has a limited resectable volume, tumor reduction and remnant liver hypertrophy are considered important strategies for making initially unresectable CRCLM resectable.

Associated liver partition and portal vein ligation for staged hepatectomy (ALPPS), first conducted in 2012, induces rapid liver growth within a short period and has a significant influence on treatment strategies for liver cancer. ALPPS has recently been reported to be useful in patients with CRCLM and insufficient future liver remnant (FLR) volume after curative liver resection. Resectability after ALPPS is very high, and few cases of insufficient remnant liver hypertrophy following ALPPS have been reported.

Herein the clinical features—including image findings and postoperative course—of a patient who underwent additional portal vein embolization following ALPPS to obtain sufficient FLR are presented.

## Case presentation

**Fig. 1:**
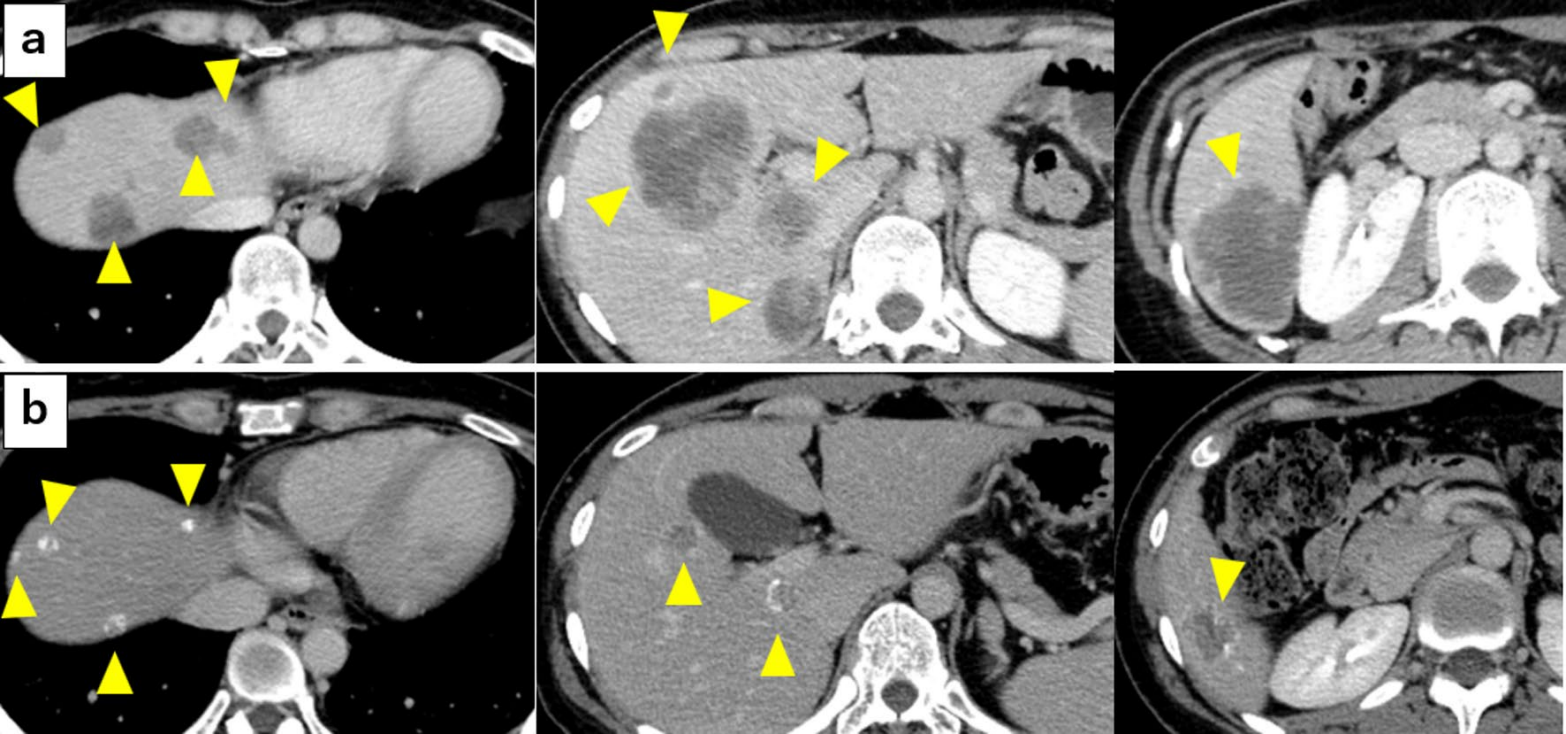
Abdominal CT findings before and after chemotherapy (15 cycles of FOLFOXIRI + bevacizumab). Yellow arrowheads indicate multiple liver metastases (**a**). After chemoradiotherapy, the liver metastases were significantly reduced in size and were calcified (**b**)

A 29-year-old woman complaining of bloody stool was referred to our hospital for evaluation and treatment. She was diagnosed with rectal cancer (Rb) with synchronous multiple liver (mainly in liver segments 2, 4a, 4b, 5, 6, and 7) and lung metastases (Fig. 1). The patient initially underwent chemotherapy (FOLFOXIRI + bevacizumab, 15 courses), which she completed with a satisfactory response (downsizing and calcification). Finally, she was diagnosed with rectal cancer Rb (ycT4bN3H2M1bPul2, Stage IVb) and referred to our department for curative resection.

**Fig. 2:**
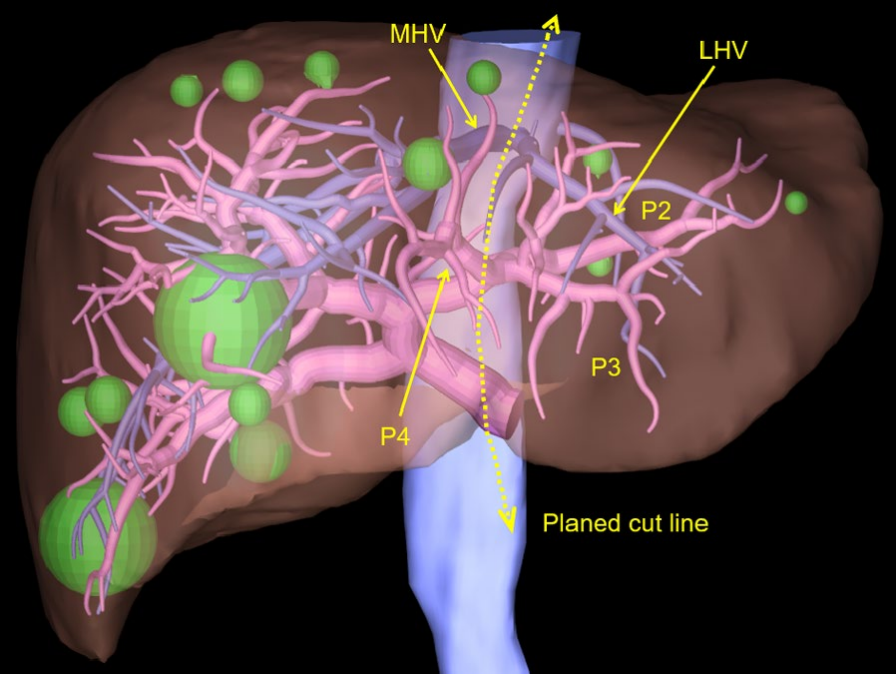
Three-dimensional reconstruction volumetry analysis showed that right trisectionectomy and partial resection of segment 2 were necessary to achieve hepatic clearance

Based on imaging study findings, right trisectionectomy was necessary to achieve hepatic clearance (Fig. 2). However, the FLR volume was calculated as only 19.2% (275 ml) of the total liver volume. Even though the patient’s liver function was normal, the indocyanine green retention rate at 15 min (ICGR15) was 3.7%, showing that radical liver resection could not be safely performed because the planned FLR volume was insufficient.

**Fig. 3:**
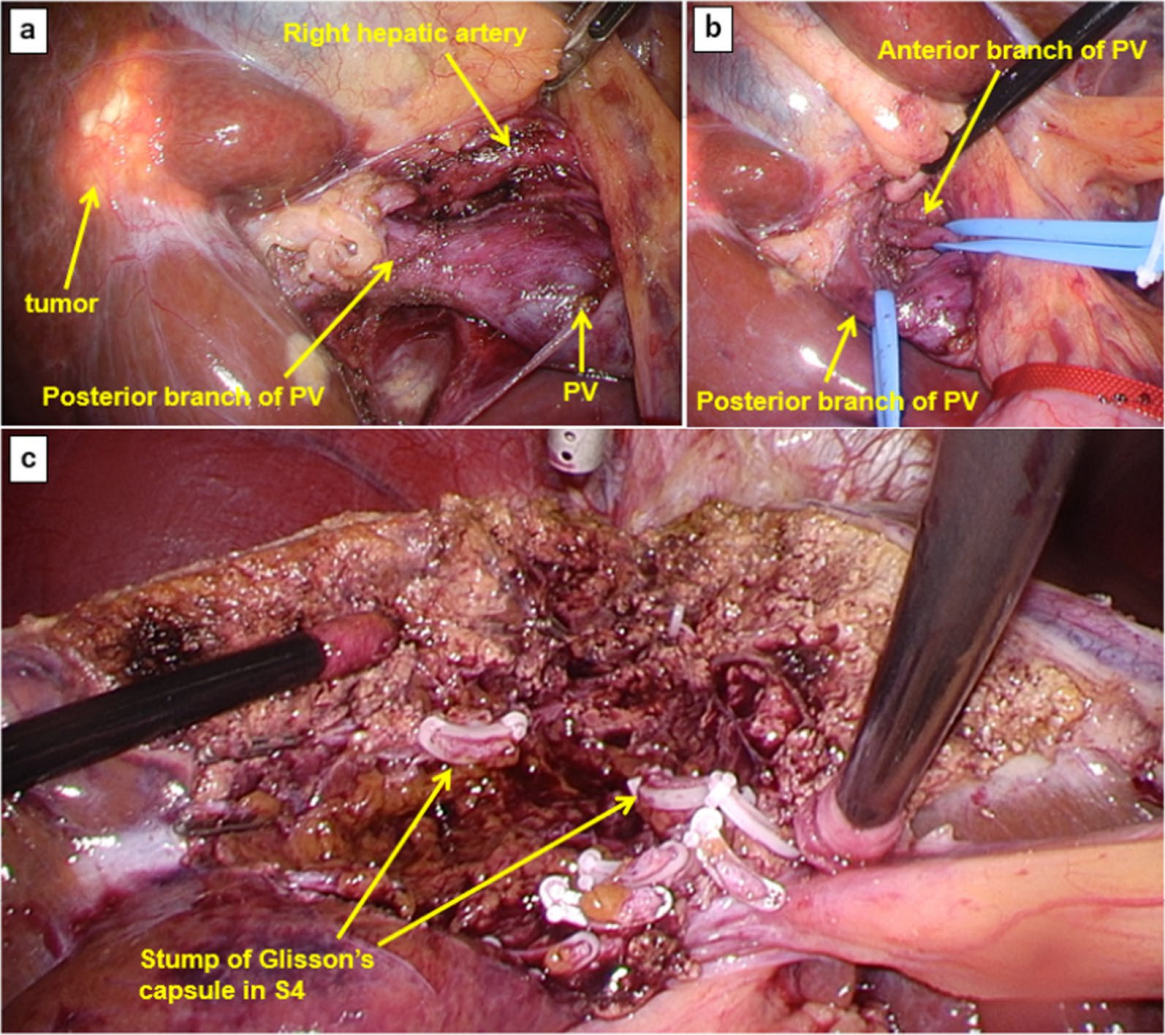
Intraoperative findings of totally laparoscopic ALPPS procedure. The hepatoduodenal ligament was dissected, and the right portal vein was identified (**a**). The posterior and anterior branches were separated and secured (**b**). **c** The liver parenchyma was transected along the right side of the umbilical portion. The Glisson’s capsule in S4 was dissected

**Fig. 4:**
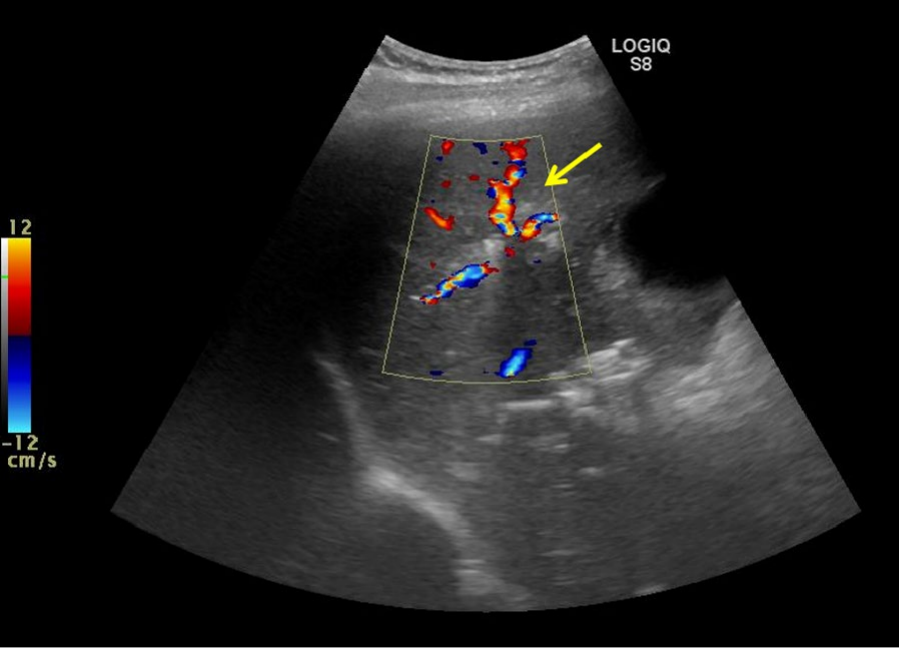
Postoperative abdominal ultrasonography after an ALPPS procedure showed complete disruption of the right portal venous blood flow, and only arterial blood flow (yellow arrow) was identified

Therefore, we decided to perform laparoscopic ALPPS to obtain sufficient and rapid hypertrophy, followed by concurrent open abdominoperineal resection. The hepatoduodenal ligament was dissected, and the right portal vein was identified (Fig. 3). The anterior and posterior branches of the right portal vein were subsequently separated. Under in-flow occlusion using the Pringle maneuver, the liver parenchyma was transected along the right side of the umbilical portion in an incision manner from the bottom to the top. The Glisson’s capsule in S4 of the liver was dissected. The anterior and posterior branches of the right portal vein were ligated. Following the laparoscopic ALPPS procedure, open abdominoperineal resection was performed. Postoperative abdominal ultrasonography immediately after ALPPS showed complete disruption of right portal venous blood flow, and that left portal venous blood flow was maintained as planned (Fig. 4).

**Fig. 5:**
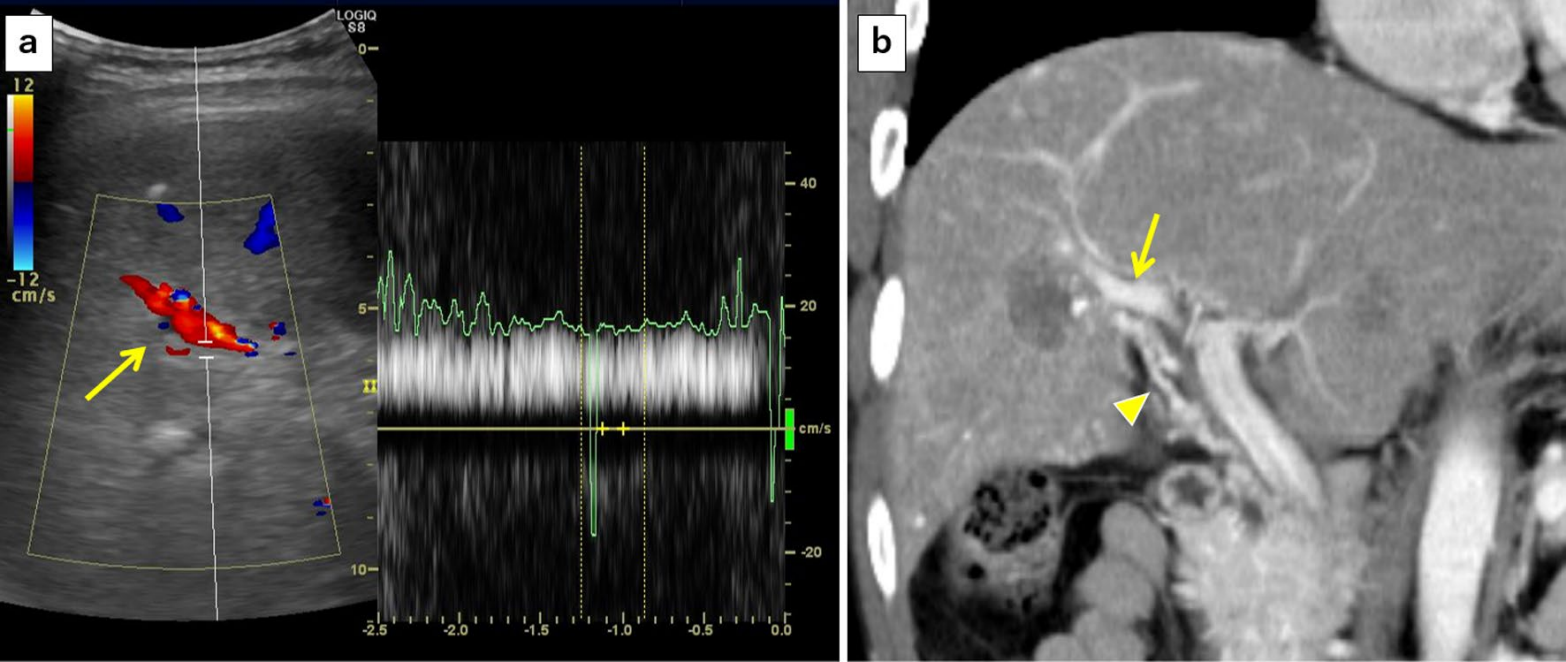
Abdominal ultrasonography (**a**) and contrast-enhanced CT (**b**) showed resumption of right portal venous blood flow (yellow arrow) via newly formed collateral vessels (yellow arrowhead) around the right Glissonean pedicle

Volumetric assessment using SYNAPSE VINCENT (Fujifilm Co Ltd, Japan) after the ALPPS showed that the ratio of FLR to total liver volume increased from 19.2 to 30.5% (399 ml) on postoperative day (POD) 7, to 32.1% (434 ml) on POD 12, and to 32.1% (434 ml) on POD 19. The FLR increase was slow, particularly after POD 7, and FLR did not attain the required volume for right trisectionectomy as radical liver resection. Abdominal ultrasonography and computed tomography (CT) on POD 19 showed resumption of right portal venous blood flow via newly formed collateral vessels around the right Glissonean pedicle, which was considered to interfere with hypertrophy of the FLR and atrophy of the right hemiliver to be resected (Fig. 5).

**Fig. 6:**
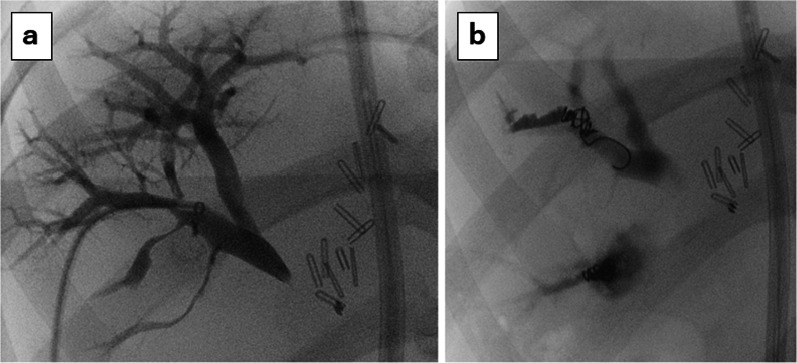
Percutaneous transhepatic portal vein embolization (PTPE) of anterior (**a**) and posterior (**b**) branches of the portal vein

**Fig. 7:**
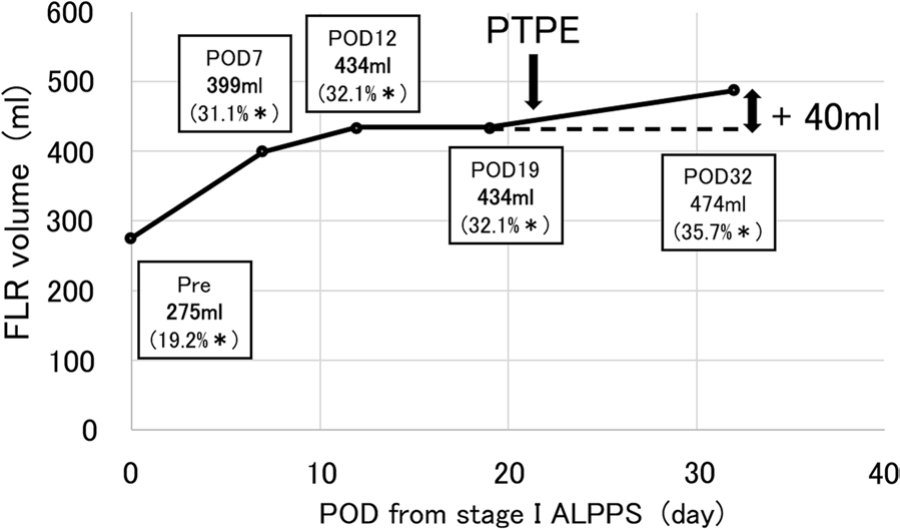
Changes over time in FLR volume after ALPPS. Rapid increase in FLR volume occurred after rescue PTPE. *: FLR volume/total liver volume ratio

We attempted to induce further liver growth by blocking portal venous blood flow to the right lobe using portal vein embolization (PVE). Additional percutaneous transhepatic portal vein embolization (PTPE) of the anterior and posterior branches of the right portal vein was performed via the 7th and 9th intercostal spaces on POD 21 (Fig. 6). Postoperative abdominal ultrasonography showed disruption of blood flow in the anterior and posterior branches of the portal vein. On POD 32, computed CT showed that the ratio of FLR to total liver volume had increased from 32.1 to 35.7% (474 ml), with an actual FLR volume increase of 40 ml (Fig. 7). Rapid increase in FLR volume was obtained after rescue PTPE.

**Fig. 8:**
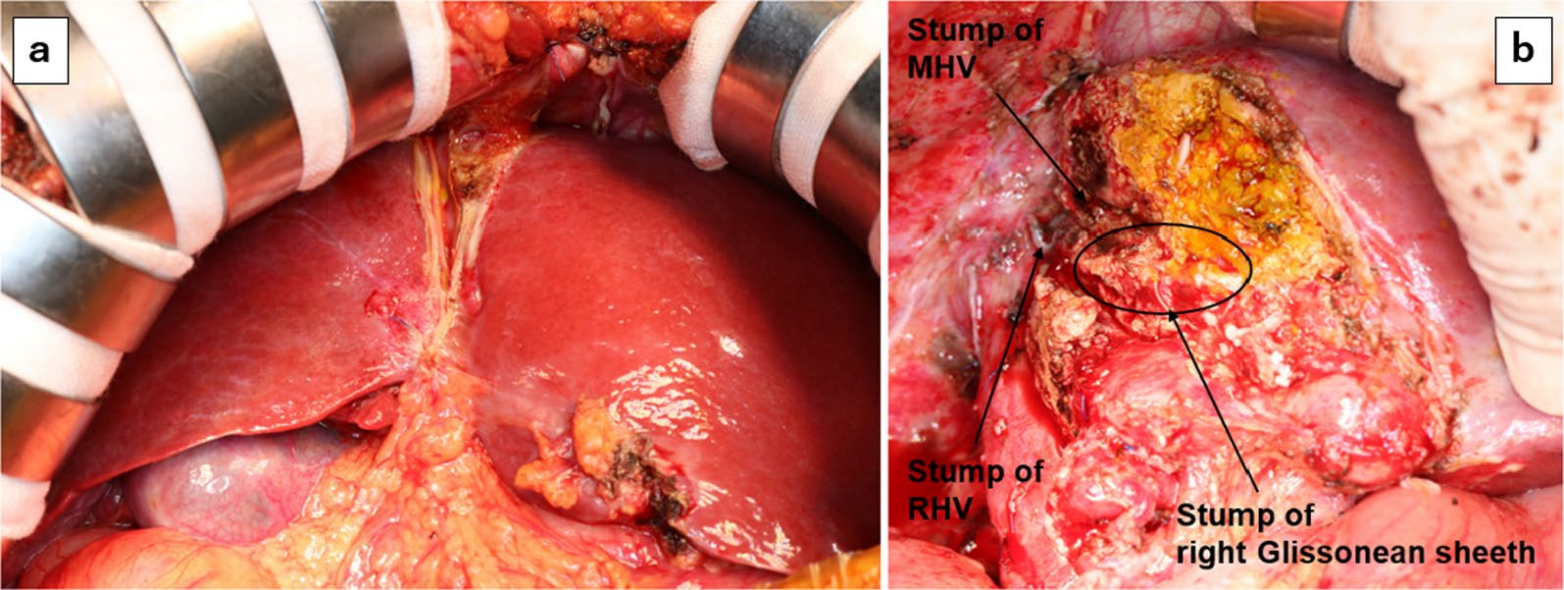
Intraoperative findings of radical hepatectomy show significant expansion of the left lateral section of the liver (**a**). Right trisectionectomy was performed (**b**)

On the 35th day after the initial surgery, the patient underwent right trisectionectomy and partial resection of S2 as radical resection of liver metastases. Exploration revealed no ascites, no peritoneal implants, new adhesion under the liver, and obvious enlargement of the left lateral section. Due to extensive adhesion and small collateral blood vessels around the right Glissonean pedicle, exposure of the anterior and posterior branches of the right portal vein was difficult. The right Glissonean pedicle was divided using a linear stapler during an en bloc procedure. Subsequently, the right and middle hepatic veins were transected. Right trisectionectomy was accomplished through the remaining liver parenchymal transection along the surface of the inferior vena cava (Fig. 8). Partial resection of S2 was subsequently performed. All surgical margins were free, and the patient was discharged on postoperative day 13 without postoperative liver failure. Thereafter, the patient underwent a thoracoscopic right lung S3 sectional resection and is currently undergoing postoperative chemotherapy.

## Discussion

Surgical resection is the only potentially curative treatment option for patients with primary or metastatic hepatic malignancies, particularly if extrahepatic tumor manifestation is absent. Hepatectomy is considered the only way to completely cure CRCLM [1–3]. Significant advancements of perioperative management in recent years have made it possible to perform even major hepatectomy with relatively low morbidity and mortality. FLR volume is a critical limiting factor when major liver resections are being planned; this is particularly true in cases of insufficient FLR volume, which is a significant risk factor for postoperative liver failure.

Several strategies have been reported to improve resectability in patients undergoing major hepatectomy. Patients with insufficient FLR volume have often undergone PVE, portal vein ligation (PVL), or two-stage hepatectomy [4–7]. PVE followed by resection is a well-established tool in small-for-size settings in liver surgery [8, 9]. Numerous studies have found that PVE and PVL increase the FLR from 10 to 46% within 2–8 weeks, and allow surgeons to perform an R0-resection in 70–100% of selected cases [10–13]. However, PVE may not induce sufficient FLR hypertrophy due to slow growth of hepatic tissue, and tumors on the non-embolized area may progress steadily during the long waiting period. The causes of insufficient liver hypertrophy following PVE have been thoroughly researched and discussed in the literature; however, they still contribute to a failure rate of approximately 20% of all procedures.

ALPPS is a new procedure which has been reported to provide a chance of curative resection for patients who were previously considered as having unresectable tumors due to small FLR volume [14]. Rapid and significant increase in FLR regeneration has been observed to be promoted by portal vein occlusion, interruption of intrahepatic vascular flow to the associated part of the liver, and inflammation at the hepatectomy site. This procedure has been proven to greatly increase FLR volume in a short period and may overcome the limitations of PVE. By using this procedure, FLR was reported to increase by 70–90% in approximately 1 week [14–18]. Petrowsky et al. reported that patients with primarily unresectable CRCLM treated by ALPPS not only have a low perioperative mortality rate, but also have a good long-term oncologic outcome, particularly in those patients with favorable tumor biology and a good response to chemotherapy [19].

However, ALPPS has several disadvantages, such as high mortality and morbidity rates. Severe complications, for instance, sepsis, liver failure, and bile leakage, have been reported. It has been reported that resectability after ALPPS procedure is extremely high and that most patients (96%) completed radical major hepatectomy following ALPPS, and there have been few reports about insufficient liver hypertrophy following ALPPS [19, 20]. Truant et al. reported the first systematic analysis of the perioperative course of ALPPS to identify factors associated with morbidity and mortality [21]. The authors stated that the failure rate after ALPPS was 5%, which could be caused by insufficient FLR, portal vein thrombosis, and interstage death, and that more than half of the patients had their intended surgery delayed beyond day 9.

In this patient, we decided to perform the ALPPS procedure to allow for complete resection of multiple and bilateral liver metastases. Unfortunately, there was slow and limited hypertrophy of the FLR, which was thought to be because of resumption of the right portal venous blood flow though newly developed collateral vessels around the Glissonean pedicle after ALPPS. Additional PVE facilitated further increase of the FLR volume and successfully salvaged ALPPS. It is hard to prove collateral vessels interfere with hypertrophy of the FLR, but rapid increase in FLR volume was obtained after rescue PTPE. It is extremely important to evaluate the liver to be resected during radical hepatectomy because of the potential for resumption of portal venous blood flow through collateral flow. If needed, additional PVE should be performed quickly. If the FLR after radical hepatectomy is insufficient, finding a way to enlarge the remnant liver is required. To achieve additional improvement in the ALPPS procedure, close attention to the anatomy and liver function in individual cases is necessary.

Some techniques have been reported to improve the results of ALPPS. Sakamoto et al. described ALPTIPS (partial liver partition until the middle hepatic vein + intraoperative transileocecal PVE) [22]. Portal vein ligation, which is performed with conventional ALPPS procedures, is associated with a risk of portal blood flow resumption from collateral vessels, as occurred in this case. Using PVE to completely occlude the intrahepatic branch of the portal vein of the part of the liver to be resected may be a useful procedure for improving ALPPS. An interesting report by Wang et al. noted that salvage trans-arterial embolization should be considered in patients with a huge HCC and chronic liver disease in cases of insufficient hypertrophy of the FLR after ALPPS [23]. How well the blood in-flow on the side of liver to be resected is controlled without complications may be a critical factor in further improving the resectability of tumors following the ALPPS operation.

## Conclusion

As described in this report, we encountered a case in which there was an insufficient increase in FLR volume due to resumption of right portal venous blood flow via newly developed collateral vessels around the right Glissonean pedicle after an ALPPS procedure. We were able to successful increase the FLR by subsequently performing PTPE, and we were then able to safely complete the intended radical major hepatectomy. Awareness of remnant portal venous blood flow through collateral blood vessels following ALPPS is important, and early additional PVE could be effective if performed.

## Data Availability

All data generated or analyzed during this study are included in the published article.

## Abbreviations

CRC: Colorectal cancer
CRCLM: Colorectal cancer liver metastasis
ALPPS: Associated liver partition and portal vein ligation for staged hepatectomy
FLR: Future liver remnant
PVE: Portal vein embolization
PTPE: Percutaneous transhepatic portal vein embolization
PVL: Portal vein ligation
